# Supplementary material for: PPARγ Targets-Derived Diagnostic and Prognostic Index for Papillary Thyroid Cancer
Source: Cancers (Basel). 2021 Oct 12;13(20):5110. doi: 10.3390/cancers13205110 (PMC8533916; doi:10.3390/cancers13205110)
Supplement: Supplementary file 1 [file cancers-13-05110-s001.zip › suppl figures.pdf]

PPARγ Targets-Derived Diagnostic and Prognostic Index for Papillary Thyroid Cancer

Jaehyung Kim, Soo Young Kim, Shi-Xun Ma, Seok-Mo Kim, Su-Jin Shin, Yong Sang Lee, Hojin Chang, Hang-Seok Chang, Cheong Soo Park and Su Bin Lim

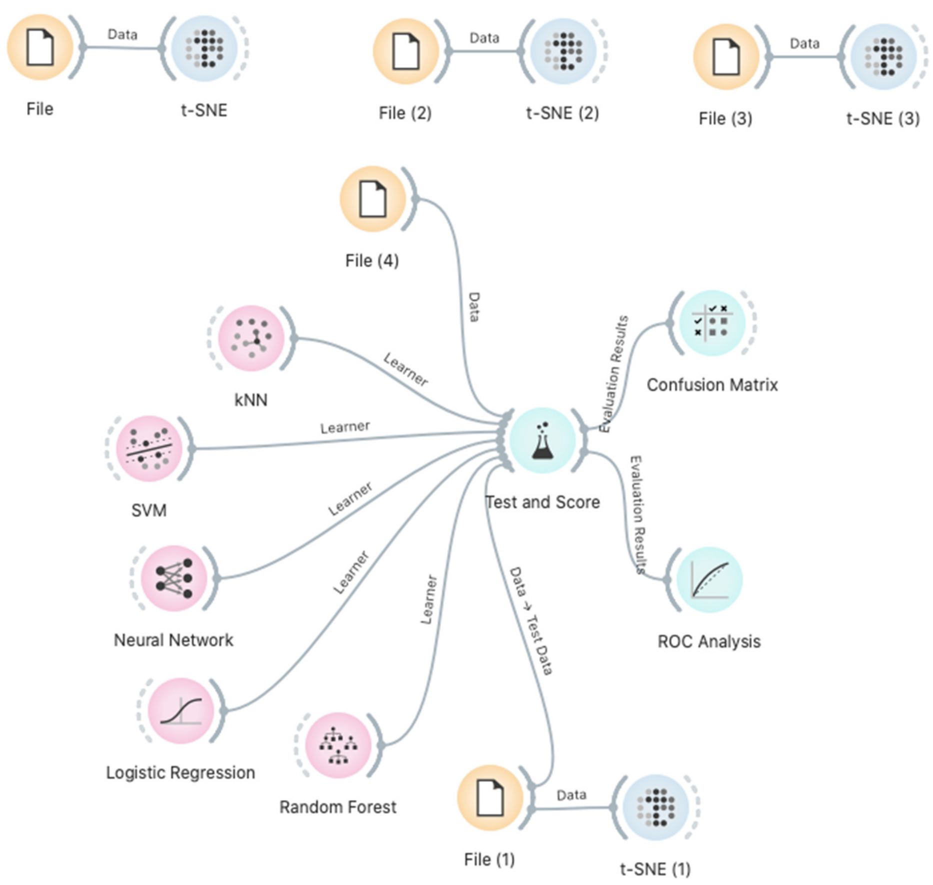

Figure S1. Orange-generated schematic workflow designed for the assessment of ML models.

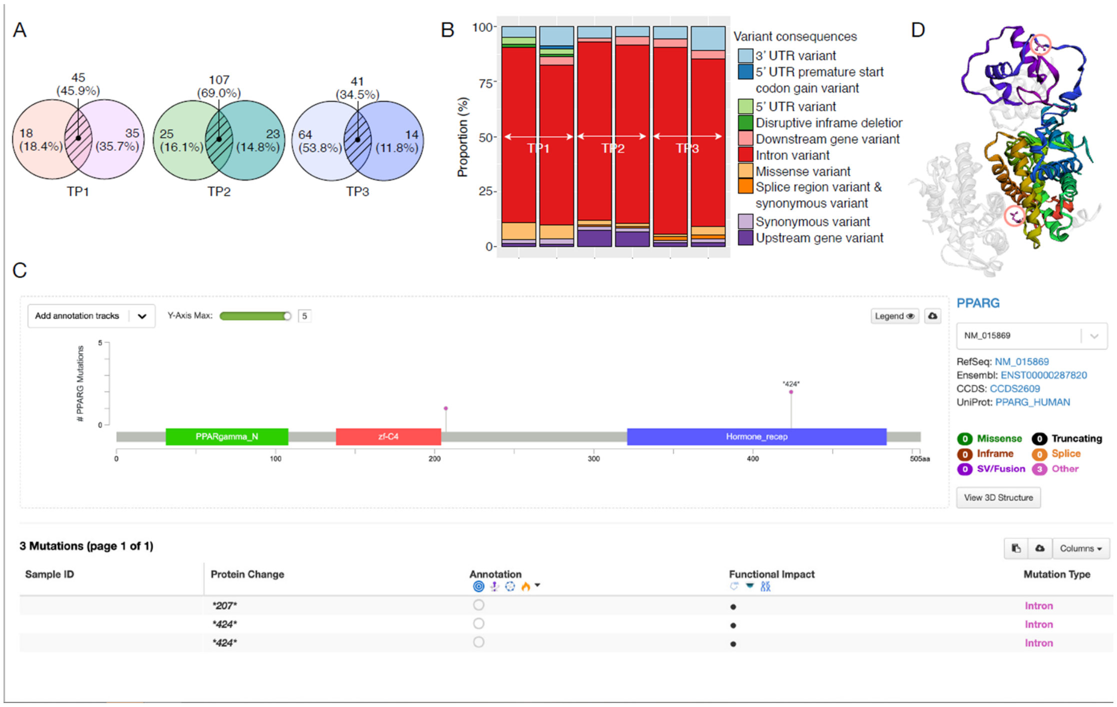

Figure S2. Targeted sequencing of thyroid-cancer related genes in monozygotic twins with PTC. (**A**) Venn diagrams showing the number of the identified variants predicted by SnpEff (v4.3) for pairwise comparison in twins (TP1–3). (**B**) Stacked bar chart showing the distribution (%) of the variant consequences. (**C**) Lollipop plots generated by MutationMapper at cBioPortal representing the variants found in PPARG. The x-axis and y-axis show the amino acid number and the frequency of the mutation, respectively. (**D**) 3D protein structure of the PPARγ-RXRα nuclear receptor complex (PDB identifier: 3E00) generated by MutationMapper at cBioPortal. The protein is colored with a rainbow gradient from red (N-terminus) to blue (C-terminus).

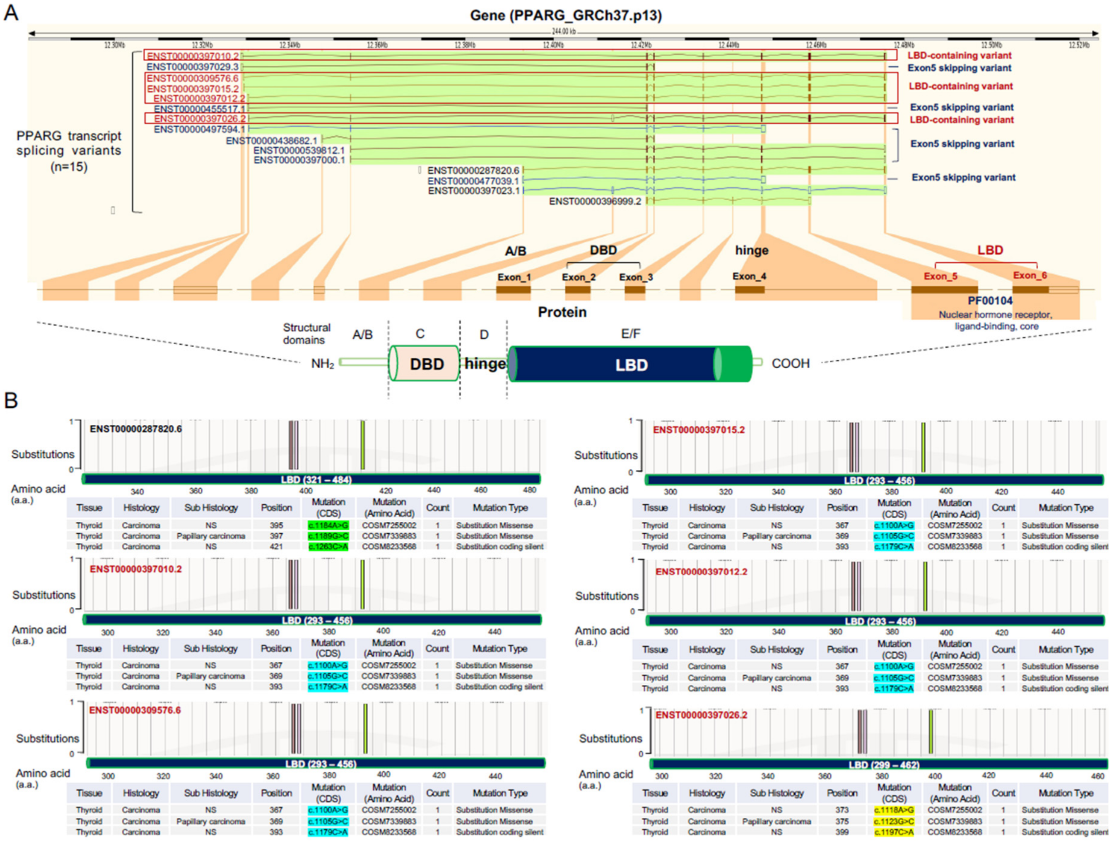

Figure S3. Transcript variants of PPARG. (**A**) Modified Ensembl-generated view of transcript variants and protein domains of *PPARG*. (**B**) CDS mutations found within LBD of transcript variants of PPARG in thyroid cancer patients using COSMIC. The horizontal axis represents the amino acid position of LBD. The vertical axis represents the number of CDS mutations occurred within the LBD. Table (below) displays a table of mutated sample, with tissue, histology, sub-histology, position, CDS mutation, count, and mutation type information.

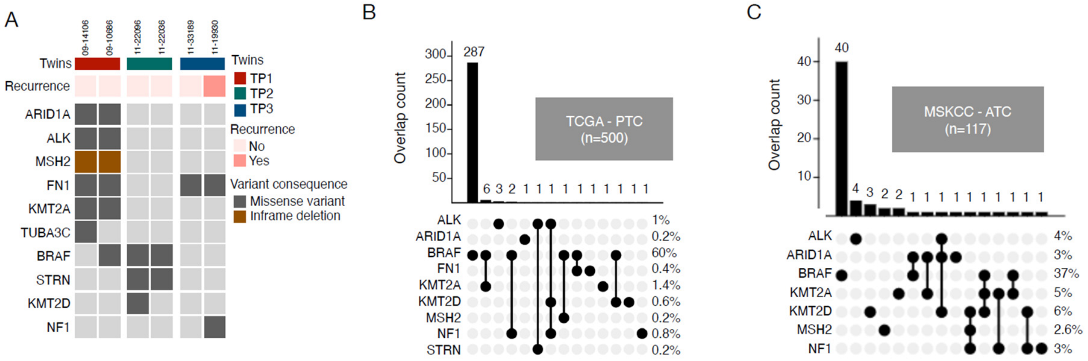

Figure S4. Non-synonymous variants found in monozygotic twins with PTC. (**A**) Genes harboring moderate impact variants (missense variants and disruptive in-frame deletion) in twins (TP1–3). The number and proportion of non-synonymous variants found in (**B**) TCGA-THCA and (**C**) MSKCC-ATC.

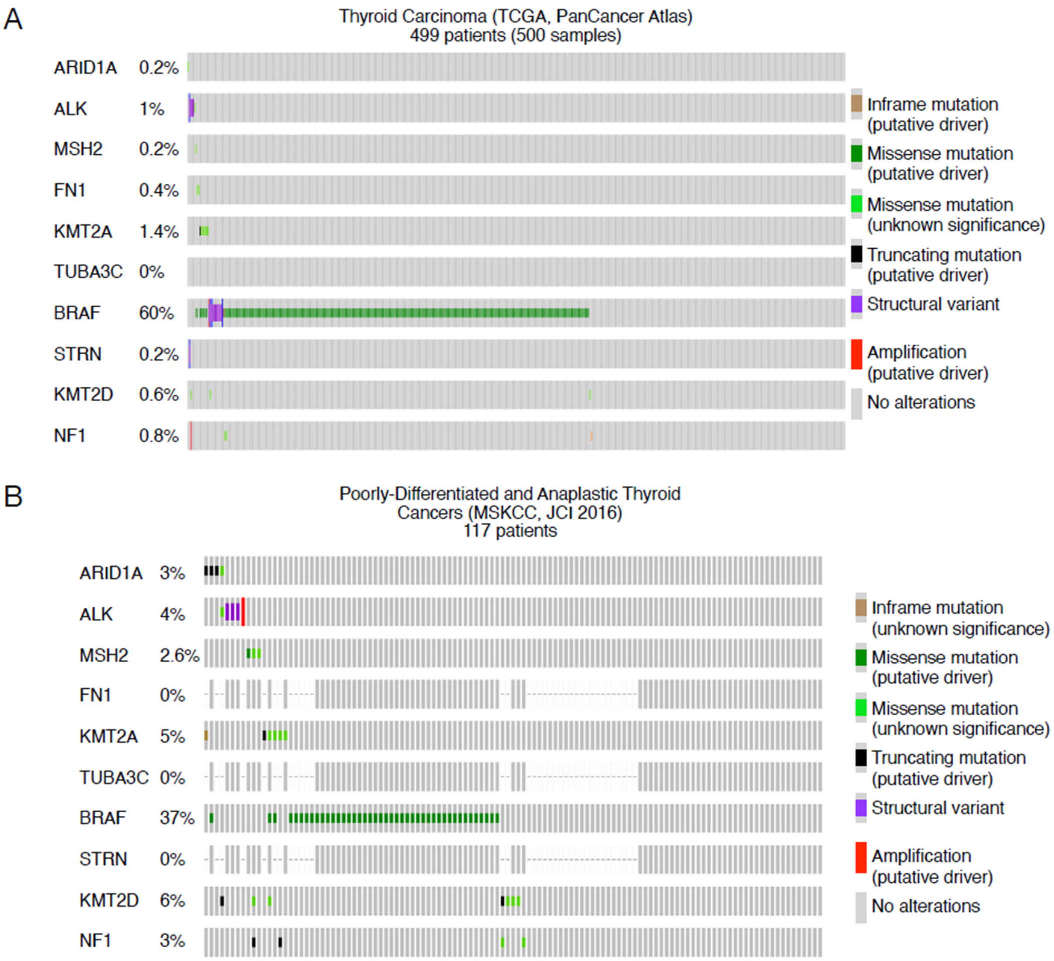

Figure S5. Types of non-synonymous variants found in (**A**) TCGA-THCA and (**B**) MSKCC-ATC.

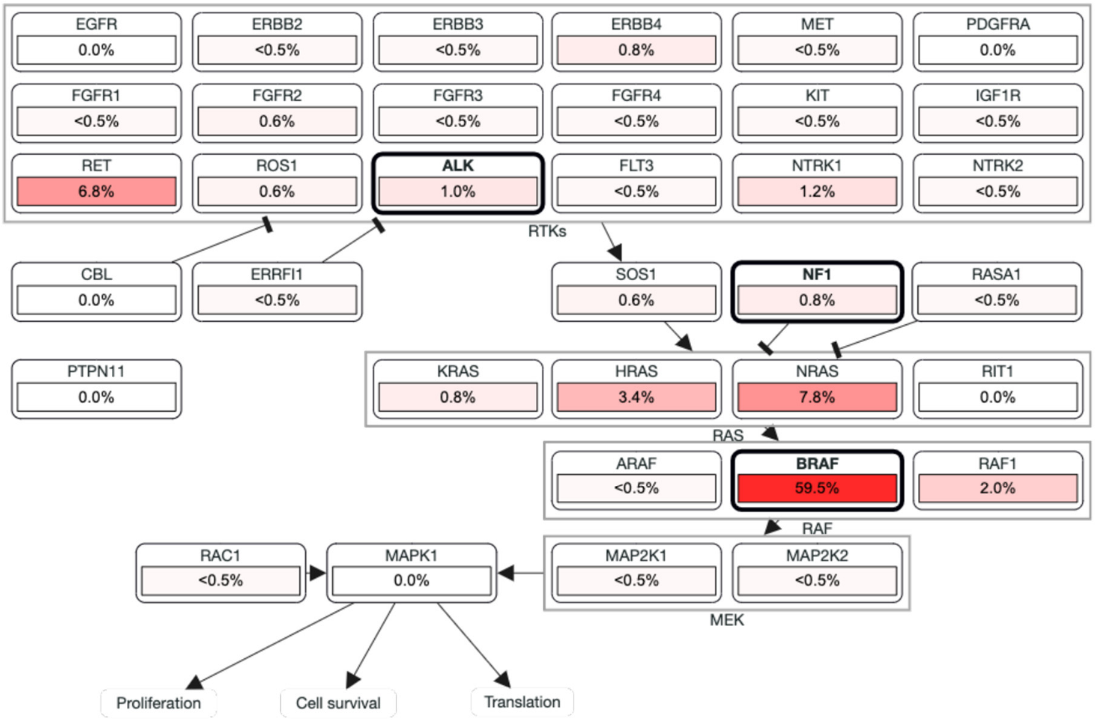

Figure S6. Schematic of the RTK-RAS pathway showing the associated genes and the proportion with the variants found in TCGA generated by the PathwayMapper tool at cBioPortal. .
